# PpZAT5 suppresses the expression of a B-box gene *PpBBX18* to inhibit anthocyanin biosynthesis in the fruit peel of red pear

**DOI:** 10.3389/fpls.2022.1022034

**Published:** 2022-10-11

**Authors:** Lu Zhang, Ruiyan Tao, Simai Wang, Yuhao Gao, Lu Wang, Shulin Yang, Xiao Zhang, Wenjie Yu, Xinyue Wu, Kunfeng Li, Junbei Ni, Yuanwen Teng, Songling Bai

**Affiliations:** ^1^ Department of Horticulture, Zhejiang University, Hangzhou, China; ^2^ Zhejiang Provincial Key Laboratory of Integrative Biology of Horticultural Plants, Zhejiang University, Hangzhou, China; ^3^ The Key Laboratory of Horticultural Plant Growth, Development and Quality Improvement, Ministry of Agriculture of China, Hangzhou, China; ^4^ Agricultural Experiment Station, Zhejiang University, Hangzhou, China

**Keywords:** light, anthocyanin biosynthesis, PpBBX18, C2H2-type zinc finger proteins, PpZAT5, *Pyrus pyrifolia*, red pear

## Abstract

BBX (B-box) proteins play a vital role in light-induced anthocyanin biosynthesis. PpBBX18 was an indispensable regulator for the induction of anthocyanin biosynthesis in the peel of red pear fruit (*Pyrus pyrifolia* Nakai.). However, the upstream regulation of BBX genes has not been well characterized. In this study, PpZAT5, a cysteine2/histidine2-type transcription factor, was discovered as the upstream negative regulator of PpBBX18. The results showed that PpZAT5 functions as a transcriptional repressor and directly binds to the CAAT motif of *PpBBX18* and inhibits its expression. *PpZAT5* expression was inhibited by light, which is converse to the expression pattern of anthocyanin-related structural genes. In addition, less anthocyanin accumulated in the *PpZAT5-*overexpressing pear calli than in the wild-type pear calli; on the contrary, more anthocyanin accumulated in PpZAT5-RNAi pear calli. Moreover, the crucial genes involved in light-induced anthocyanin biosynthesis were markedly down-regulated in the transcriptome of PpZAT5 overexpression pear calli compared to wild-type. In conclusion, our study indicates that *PpBBX18* is negatively regulated by a C2H2-type transcriptional repressor, PpZAT5, which reduces anthocyanin content in pear. The present results demonstrate an upstream molecular mechanism of *PpBBX18* and provide insights into light-induced anthocyanin biosynthesis.

## Introduction

Although the pear cultivars with the peel color of green, yellow, and brown are still dominant in the Chinese pear market, red Asian pears are gradually gaining market acceptance and consumer preference (Zhang et al., 2013; Sun et al., 2014). The red coloration of pear is attributed to anthocyanin accumulation, and anthocyanin has been identified as the main pigment in many fruits. Anthocyanin is synthesized by the phenylpropanoid pathway, in which PAL (phenylalanine ammonia-lyase), CHS (chalcone synthase), CHI (chalcone isomerase), F3H (flavanone 3-hydroxylase), F3’H (flavonoid 3’-hydroxylase), DFR (dihydroflavonol 4-reductase), ANS (anthocyanin synthase), and UFGT (UDP-glucose: flavonoid 3-glucosyltransferase) are the critical enzymes encoded by structure genes (Winkel-Shirley, 2001; Koes et al., 2005). R2R3-MYB, basic helix-loop-helix (bHLH), and WD40-repeat protein (WDR) regulate the expression of structural genes in the process of anthocyanin biosynthesis, and they frequently exert efficacy as the MYB-bHLH-WDR (MBW) complex (Koes et al., 2005; Jaakola, 2013; Xu et al., 2015). A series of MYB proteins related to anthocyanin biosynthesis have been identified, such as *AtMYB75* (*PAP1*), and *AtMYB90* (*PAP2*) in Arabidopsis (Borevitz et al., 2000; Allan and Espley, 2018), *MdMYB1/A/10* in apple (Takos et al., 2006; Ban et al., 2007; Espley et al., 2007) and *PpMYB10*, *PpMYB114* in pear (Feng et al., 2010; Yao et al., 2017).

Among the three components of the MBW complex, the expression of *MYB* is the most significantly regulated by various internal and external factors at both the transcriptional and translational levels, among which light is considered as the indispensable environmental factor in many plant species (Allan and Espley, 2018). In the light-signal transduction cascade, ELONGATED HYPOCOTYL 5 (HY5) is the hub transcription factor integrating various upstream light signals and regulating the expression of downstream genes, including the anthocyanin biosynthetic genes (Shin et al., 2013; An et al., 2017; Bai et al., 2019b). However, due to the lack of transcription activation motifs, HY5 proteins usually form a complex with other transcription factors. Recent studies showed that a series of BBX proteins serve as the partners of HY5 proteins, forming complex and regulating the downstream genes. In pear, PpHY5 interacts with PpBBX18 (the ortholog of Arabidopsis AtBBX21) and PpBBX16 (the ortholog of Arabidopsis AtBBX22) and activates the expression of *PpMYB10*. PpBBX21 (the ortholog of Arabidopsis AtBBX24/25) could interfere with forming the active transcription activator complex PpHY5-PpBBX18 and then suppress anthocyanin biosynthesis (Bai et al., 2019b).

The expressions of BBX proteins are usually highly activated by light. In Arabidopsis, the expression of *LZF1/AtBBX22* is upregulated by light and largely depends on HY5 (Chang et al., 2008). Light dynamically promotes the expression of *AtBBX21* at both the transcription and translation levels (Xu et al., 2016). Similarly, most *BBX*s related to anthocyanin biosynthesis are activated by light. Our previous research showed that *PpBBX18* and *PpBBX21* are upregulated under light conditions and then regulate anthocyanin biosynthesis (Bai et al., 2019b). However, the influence of light on anthocyanin biosynthesis is a fine-tuned process (Jaakola, 2013), so we speculate that the release of transcriptional suppression in light conditions might also be involved in the expression of *BBX* genes and further regulate anthocyanin biosynthesis.

This study applied yeast one-hybrid library screening with *PpBBX18* promoter as the bait to elucidate the transcriptional regulation of PpBBX18. As a result, the *PpZAT5* was obtained, its expression was inhibited by light. ZINC FINGER of ARABIDOPSIS THALIANA (ZAT) proteins belong to cysteine2/histidine2-type (C2H2-type) zinc finger protein family, harboring a motif with paired cysteine and histidine residues which together chelate a zinc ion to stabilize the domain (Shimeld, 2008). In general, the C2H2 zinc finger motif ranges in length from 25 to 30 residuals with the pattern of amino acids X-X-C-X(1–5)-C-X(12)-H-X(3–6)-H (X may be any amino acid and the numbers in brackets show the number of residues) (Shimeld, 2008). Besides zinc finger domains, a majority of C2H2 zinc finger proteins have nuclear localization signal (NLS, also called B-box), an EAR motif [L/FDLNL/F(x)P] locating at the C terminus with repression activity and L-box related to protein-protein interaction (Ciftci-Yilmaz and Mittler, 2008). In addition, most C2H2 zinc finger proteins contain a highly conserved plant-specific motif, QALGGH, which plays a critical role in DNA binding activity (Kubo et al., 1998; Takatsuji, 1998).

In *Arabidopsis thaliana*, 176 C2H2 zinc finger members have been identified and classified into three different sets (A, B, and C) according to the positions of the zinc finger motif and their sequences (Englbrecht et al., 2004). C2H2 zinc finger proteins play an essential role in the regulation of metabolic pathways of plants, particularly in stress response and defense activation (Englbrecht et al., 2004; Ciftci-Yilmaz and Mittler, 2008). *AtZAT18* positively regulated the response to drought stress in Arabidopsis (Chan et al., 2017), whereas SLZF3 (the ortholog of Arabidopsis ZAT12) increased the accumulation of AsA and promoted the salt tolerance in tomato and Arabidopsis (Li et al., 2018). In addition, some specific C2H2-type zinc finger proteins could promote anthocyanin biosynthesis in plants. *SlZF2* (the ortholog of Arabidopsis ZAT10) positively responded to salt stress, drought, and potassium chloride treatments and facilitated the accumulation of anthocyanin and malondialdehyde in tomatoes (*Solanum lycopersicum*) (Hichri et al., 2014). MdZAT5 was induced by various abiotic stress treatments and promoted anthocyanin biosynthesis by regulating the expressions of genes in the anthocyanin biosynthesis pathway (Wang et al., 2022). *AtZAT6* participated in oxidative stress-induced anthocyanin biosynthesis in Arabidopsis by binding to the promoters of several genes associated with anthocyanin (Shi et al., 2018). Although most C2H2 zinc finger proteins regulate metabolic pathways by binding to downstream genes, the recognition site of C2H2 zinc finger protein is not entirely understood. C2H2 zinc finger protein ZPT2-2 of petunia can specifically recognize two tandemly repeated AG(C)T core sequences separated by 13 bp (Takatsuji, 1998), while Arabidopsis Di19 (one C2H2 zinc-finger protein) and AtZAT6 bind to the TACA(A/G)T element of downstream genes promoters (Liu et al., 2013; Shi et al., 2014). In pears, the binding site of C2H2 zinc finger proteins had not been reported.

In this study, we found that PpZAT5 encodes a transcriptional repressor with the EAR motif at the C terminus and its expression was suppressed by light. PpZAT5 could directly bind to CAAT motif of *PpBBX18* promoter and inhibit the anthocyanin biosynthesis by repressing *PpBBX18* expression. Our work revealed the upstream regulatory mechanism of PpBBX18, which would be helpful for elucidating the mechanism of pear coloration.

## Materials and methods

### Plant materials and treatment

Fruits were treated as previously described (Ni et al., 2020). ‘Hongzaosu’ pears *(Pyrus pyrifolia* × *Pyrus communis*) were from the orchard of the Institute of Horticulture, Henan Academy of Agricultural Sciences, Henan, China. Fruits were covered with lightproof double-layered paper bags during the growing season and bagged fruits were collected at maturity (155 days after full bloom), then immediately transported to laboratory. Bag-removed fruits were incubated for 10 days under continuous white light (60 μmol m^-2^ s^-1^) that was provided by overhead LEDs in a growth chamber set at 17°C and 80% relative humidity. After initiating the irradiation, the fruit peel was sampled at 0, 12, 24, 48, 72, 144, and 240 h. The peels of two fruits randomly sampled were collected as one biological replicate, and analyses were completed with three biological replicates (i.e., six fruits in total). The collected peels were immediately frozen in liquid nitrogen and stored at -80°C.

The pear calli induced from young fruit flesh cells of *Pyrus communis* ‘Clapp’s Favorite’ were used to transformation test as previously described (Bai et al., 2019a). In short, we constructed an expression vector with pCAMBIA1301-PpZAT5, pCAMBIA1301-PpZAT5-RNAi and pCAMBIA1300-PpZAT5 plasmid at first and then transformed them into *Agrobacterium tumefaciens* strain EHA105. Pear calli were incubated with *A. tumefaciens* cells harboring vectors (OD600 of 0.4-0.6) for 12-14 minutes and co-cultured for 2-3 days in darkness at 24°C. Pear calli were cultivated on solid Murashige and Skoog medium with proper hygromycin in the next 2 to 3 months and were sub-cultured every 2 weeks. For the treatment, both freshly sub-cultured transgenic and wild-type pear calli were exposed to continuous light until they turned red. Make sure every treatment was completed with at least three biological duplicates. The primers used for vector construction are listed in **Supplementary Table 2**.

### Anthocyanin measurement

Total anthocyanin contents of red pear peel and pear calli were measured as previously described by Ni (Ni et al., 2021). Briefly, 0.1 g powder of frozen pear calli or peels was soaked in 1mL of methanol: acetic acid (99:1, v/v) solution and incubated overnight at 4°C in darkness. And then, the absorbance of the extracting solution was measured by a DU800 spectrophotometer (Beckman Coulter, Brea, CA, USA) at 530, 620, and 650 nm. The anthocyanin content of pear calli was calculated as the following formula: ([A530-A650]-0.2×[A650-A620])/0.1.

### RNA extraction and quantitative reverse-transcription PCR

RNA of pear peel and pear calli was extracted following a modified cetyl-trimethyl-ammonium bromide method (Zhang et al., 2013). According to the instruction of PrimeScript™ RT Reagent Kit with gDNA Eraser (TaKaRa, https://www.takarabio.com/), first-strand cDNA was synthesized with 1μg RNA served as the template, and the qRT-PCR assay was conducted by a CFX Connect™ real-time PCR system (Bio-Rad, https://www.bio-rad.com/) and then adjusted cDNA concentrations to make sure that amount of template for the qPCR analysis was equal. The relative expression level of genes was calculated using the 2^-△△^ *^Ct^* method with the pear *Actin* gene (*PpActin*; GenBank accession No. JN684184) as the reference controls, and the analyses were carried out with three biological replicates. The primers used were designed by the Primer-BLAST online tool (https://www.ncbi.nlm.nih.gov/tools/primer-blast/) and are listed in **Supplementary Table 2**.

### Subcellular localization

A vector containing pCAMBIA1300-PpZAT5 plasmid and the empty vector (i.e., GFP alone) as control was constructed and then transformed into *Agrobacterium tumefaciens* strain GV3101. *A. tumefaciens* cells harboring pCAMBIA1300-PpZAT5 and empty vector were injected into *N. benthamiana* (mCherry) leaves, respectively. Fluorescence signals were detected by an A1 confocal laser scanning microscope (Nikon, Tokyo, Japan) at 36-72 h after the injection. The primers used are listed in **Supplementary Table 2**.

### Trans-acting activity assay

The full length of *PpZAT5* CDS was cloned to the pGBKT7 (BD) vector containing the VP16 fragment. And PpERF105-BD and PpERF105-VP16BD recombinant plasmid as well as positive (VP16-BD) and negative (BD) controls were transformed into yeast *(Saccharomyces cerevisiae)* strain AH109 cells independently with the Yeastmaker™ Yeast Transformation System 2 (TaKaRa). The yeast transformants were selected in plates containing a selective synthetic dextrose medium lacking tryptophan (SD/-Trp). The positive yeast transformants were further screened on plates containing SD/-Trp/-His medium supplemented with X-α-gal (SD/-Trp/-His/X-α-gal). The trans-activity of PpZAT5 was assessed according to the blue color of yeast strain AH109 growing on SD/-Trp/-His medium. The quantitative analysis of trans-activity based on β-galactosidase activity was measured as previously described by Bai (Bai et al., 2019b) with at least three biological replicates for each measurement. The primers used are listed in **Supplementary Table 2**.

### Yeast one-hybrid assay

Yeast one-hybrid assays were performed following the manufacturer’s protocol of Matchmaker Gold Yeast One-Hybrid System Kit (TaKaRa, Otsu, Japan). Promoter fragments were ligated to the pAbAi vector and linearized, while *PpZAT5* CDSs were cloned to the pGADT7 vector (AD). pAbAi vectors were transformed into the yeast strain Y1HGold and selected on plates containing a selective synthetic dextrose medium lacking uracil (SD/-Ura). And then prey vectors were transformed into Y1HGold caring pAbAi vector and detected on SD/-Leu/AbA plates. The primers used are listed in **Supplementary Table 2**.

### Dual-luciferase assay

Dual-luciferase assays were carried out as previously described (Bai et al., 2019a). The full-length CDS of *PpZAT5* was cloned to the pGreenII 0029 62-SK vector, while *PpBBX18* promoter sequence was inserted into the pGreenII 0800-LUC vector. Both vectors were transformed into *A. tumefaciens* strain GV3101, and then *A. tumefaciens* harboring pGreenII 0029 62-SK vector and pGreenII 0800-LUC vector were injected with *N. benthamiana* leaves in 10:1 ratio. The Dual-Luciferase Reporter Assay System (E710, Promega, Madison, WI, USA) was used to analyze the firefly luciferase and *Renilla* luciferase activities with Modulus Luminometers (GloMax96, Promega). The analysis was carried out in three independent experiments with at least six biological replicates per assay. The primers used are listed in **Supplementary Table 2**.

### Electrophoretic mobility shift assay

EMSAs were conducted as previously described (Bai et al., 2019b). Briefly, oligonucleotide probes labeled with biotin at the 3’ end were synthesized (Huagen, http://www.huagene.cn) and then annealed to complementary oligonucleotides (95°C for 5 min, 70°C for 20 min, and cooled it down to room temperature and set aside). Following the instructions of LightShift Chemiluminescent EMSA Kit (Thermo Scientific), both binding buffer [2.5% glycerol, 5 mM MgCl_2_, 50 ng poly (deoxyinosinic-deoxycytidylic) acid, 0.05% NP-40] with and without proteins were prepared for incubation of 30 fmol of the labeled probe at room temperature for 10 min. The non-labeled probe was added to reactions in different volumes for the competition assays. After SDS-PAGE electrophoresis, the DNA was transferred to a nylon membrane (Millipore, http://www.merckmillipore.com/), and the anti-biotin antibody provided in the kit was used for biotin-labeled probes detection.

### RNA-seq data analysis

We used the wild-type and pCambia1301-PpZAT5 pear calli for RNA-Seq analysis. The calli were incubated under dark or light for two days for RNA-Seq. The calli of three different lines were simultaneously treated and used as biological replicates. The total RNAs were extracted as described above. Ten micrograms of total RNA each were used for next-generation sequencing. Novogene (Beijing, China) performed the library construction and sequencing using the HiSeq X (Illumina, San Diego, CA) platform with a 150-bp pair-end strategy. The clean reads were mapped to the European pear genome sequence (Chagne et al., 2014) http://www.rosaceae.org) using HISAT2 (Pertea et al., 2016) with default parameters. The reads were then assembled into transcripts and compared with reference gene models using StringTie (Pertea et al., 2016). The differentially expressed gene analysis was performed using DESeq2 (Love et al., 2014).

## Results

### Identification and characterization of *PpZAT5*

The functions of BBX proteins in light-induced anthocyanin biosynthesis have been well studied, but their upstream regulatory mechanism is still unclear. To figure out the upstream gene regulatory network of PpBBX18, we established a yeast one-hybrid system with *PpBBX18* promoter as bait based on the cDNA library of red pear peel (*Pyrus pyrifolia* Nakai). Following bioinformatics sequencing and analysis, 14 cDNA sequences were identified (**Table 1**), among which *PpZAT5* was selected for further analysis since C2H2 zinc finger proteins play vital roles in regulating metabolic pathways of plants, including anthocyanin biosynthesis.

**Table 1:** Screening results of *PpBBX18* promotor transcriptional factors.

Number	NCBI Number	Annotation	Frequency
1	XP_009350033.1	PREDICTED: protein BASIC PENTACYSTEINE6- 3like [Pyrus x bretschneideri]	3
**2**	**XP_009356940.1**	**PREDICTED: zinc finger protein ZAT5 [Pyrus x bretschneideri]**	**1**
3	XP_009337640.1	PREDICTED: protein CHAPERONE-LIKE PROTEIN OF POR1, chloroplastic [Pyrus x bretschneideri]	1
4	XP_009339761.1	PREDICTED: NAC domain-containing protein 17-like [Pyrus x bretschneideri]	1
5	XP_009338942.1	PREDICTED: NEP1-interacting protein-like 2 isoform X2 [Pyrus x bretschneideri]	1
6	XP_009351493.1	PREDICTED: MLP-like protein 43 [Pyrus x bretschneideri]	1
7	XP_008380242.1	PREDICTED: serine/threonine-protein phosphatase 5- like isoform X2 [Malus domestica]	1
8	XP_009368514.1	PREDICTED: trihelix transcription factor GT-2-like [Pyrus x bretschneideri]	1
9	XP_008359462.1	PREDICTED: R3H domain-containing protein 2-like [Malus domestica]	1
10	XP_009333978.1	PREDICTED: coatomer subunit beta-1-like [Pyrus x bretschneideri]	1
11	XP_009372564.1	associated hydrolase (LOC103961701), transcript 1 variant X4, mRNA	1
12	XP_009355565.1	PREDICTED: 50S ribosomal protein L24, chloroplastic [Pyrus x bretschneideri]	1
13	XP_008380242.1	PREDICTED: serine/threonine-protein phosphatase 5- like isoform X2 [Malus domestica]	1
14	XP_008342254.1	PREDICTED: long chain acyl-CoA synthetase 8 [Malus domestica]	1

The detected genes in yeast one-hybrid library screening assay with *PpBBX18* promoter as bait. PpZAT5 is marked in bold.

**Figure 1:**
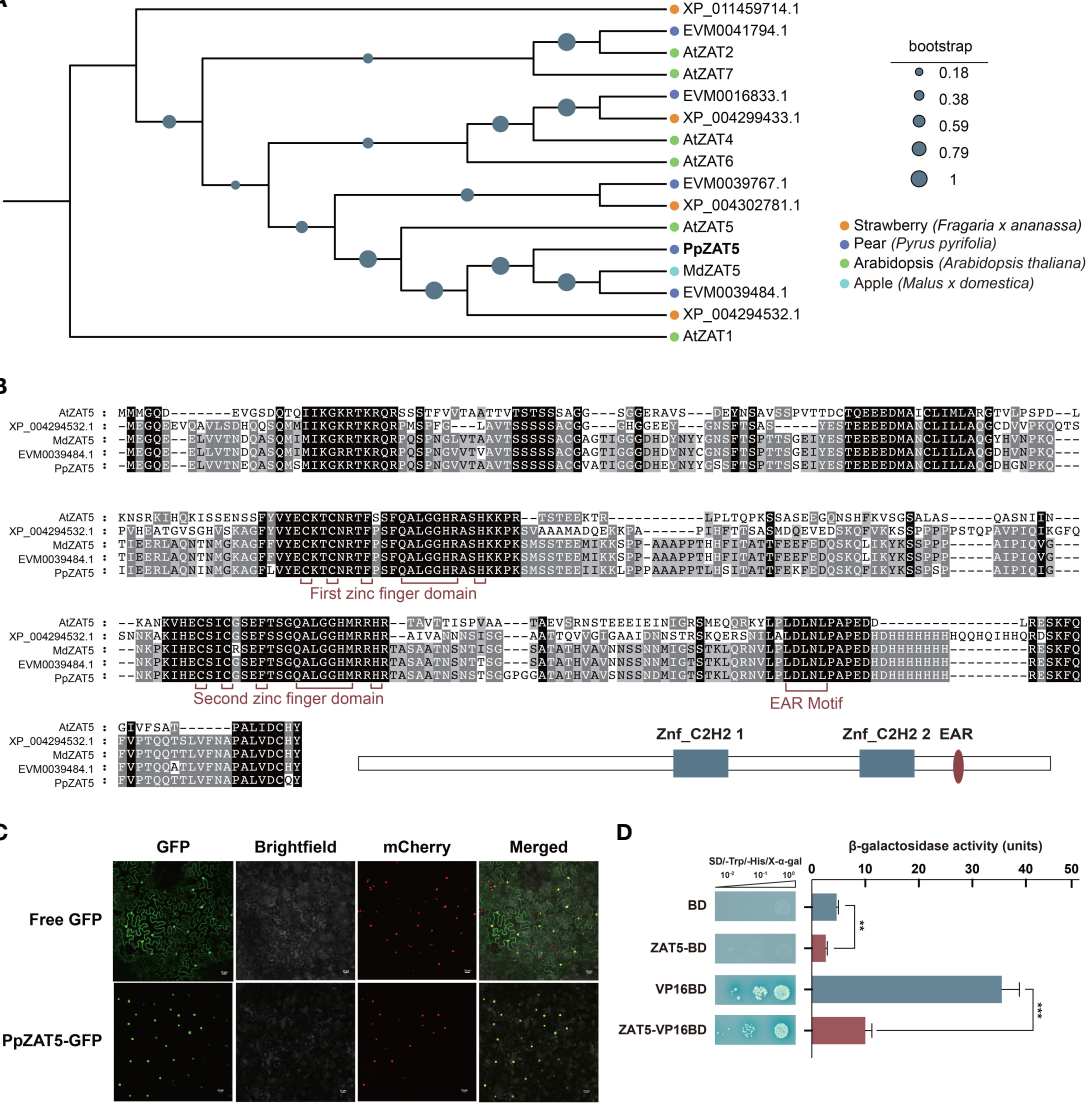
Bioinformatics analysis and trans-acting activity of PpZAT5. **(A)** Phylogenetic analysis of the C2H2-type zinc finger proteins from pear and other plant species obtained through blast algorithm using Arabidopsis ZAT1 to ZAT7 as queries. Pear (*Pyrus pyrifolia*); Apple (*Malus x domestica*): MdZAT5(MD03G1128800); Arabidopsis (*Arabidopsis thaliana*): AtZAT1(AT2G46800.1), AtZAT2(AT2G17180.1), AtZAT4(AT2G45120.1), AtZAT5(AT2G28200.1), AtZAT6(AT5G04340.1), AtZAT7(AT3G46090.1); Strawberry (*Fragaria x ananassa*). **(B)** Alignment of C2H2-type zinc finger proteins from pear, strawberry and apple. **(C)** Subcellular localization of PpZAT5 expressed in tobacco leaf cells. Scale bars = 25 μm. **(D)** Trans-acting activity of PpZAT5 transformed into yeast cells. The β-galactosidase activities reflected the trans-acting activities. Error bars for trans-acting activity represent the standard deviation of three independent experiments each. Asterisks indicate significant differences (two-tailed Student’s *t*-test, **P < 0.01, ***P < 0.001).

ZAT proteins in pear, apple, and strawberry genomes were identified using the blast algorithm using Arabidopsis ZAT1 to ZAT7 as queries and then established the phylogenetic tree. The phylogenetic analysis revealed that PpZAT5 was a homologous protein of AtZAT5 (**Figure 1A**). Moreover, multiple sequence alignment of some C2H2-type zinc finger proteins conserved domains showed that PpZAT5 contained two zinc finger conserved domains in 369 bp to 447 bp and 633 bp to 705 bp, as well as an EAR motif located at the C terminus as shown in **Figure 1B**. EAR motif that is located at the C terminus of PpZAT5 is generally related to repression activity, which suggests PpZAT5 is a candidate transcriptional repressor.

A subcellular localization assay of PpZAT5 showed that yellow fluorescent signals appeared only in the nuclei after transiently expressing PpZAT5-GFP fusion protein in the leaves of tobacco (*Nicotiana benthamiana*) plants with mCherry in the nucleus. In contrast, the fluorescence signal of GFP alone was observed throughout the cells following the expression of empty GFP protein (**Figure 1C**). We further analyzed the trans-acting activity of PpZAT5 in the yeast system. The AH109 yeast cells containing PpZAT5 fused to the DNA-binding domain (BD) failed to grow on the selective medium (SD/-Trp/-His) supplemented with X-α-gal, while the positive control with VP16-BD grew well and turned to blue. The fusion of PpZAT5 with the DNA-binding domain showed less β-galactosidase activity than that of the construct harboring only BD, and the positive control VP16BD showed higher β-galactosidase activity than the fusion of PpZAT5 and VP16BD (**Figure 1D**). Hence, PpZAT5 appears to be a nuclear-localized transcriptional repressor.

### The expression pattern of PpZAT5 is negatively related to that of anthocyanin biosynthetic genes

**Figure 2:**
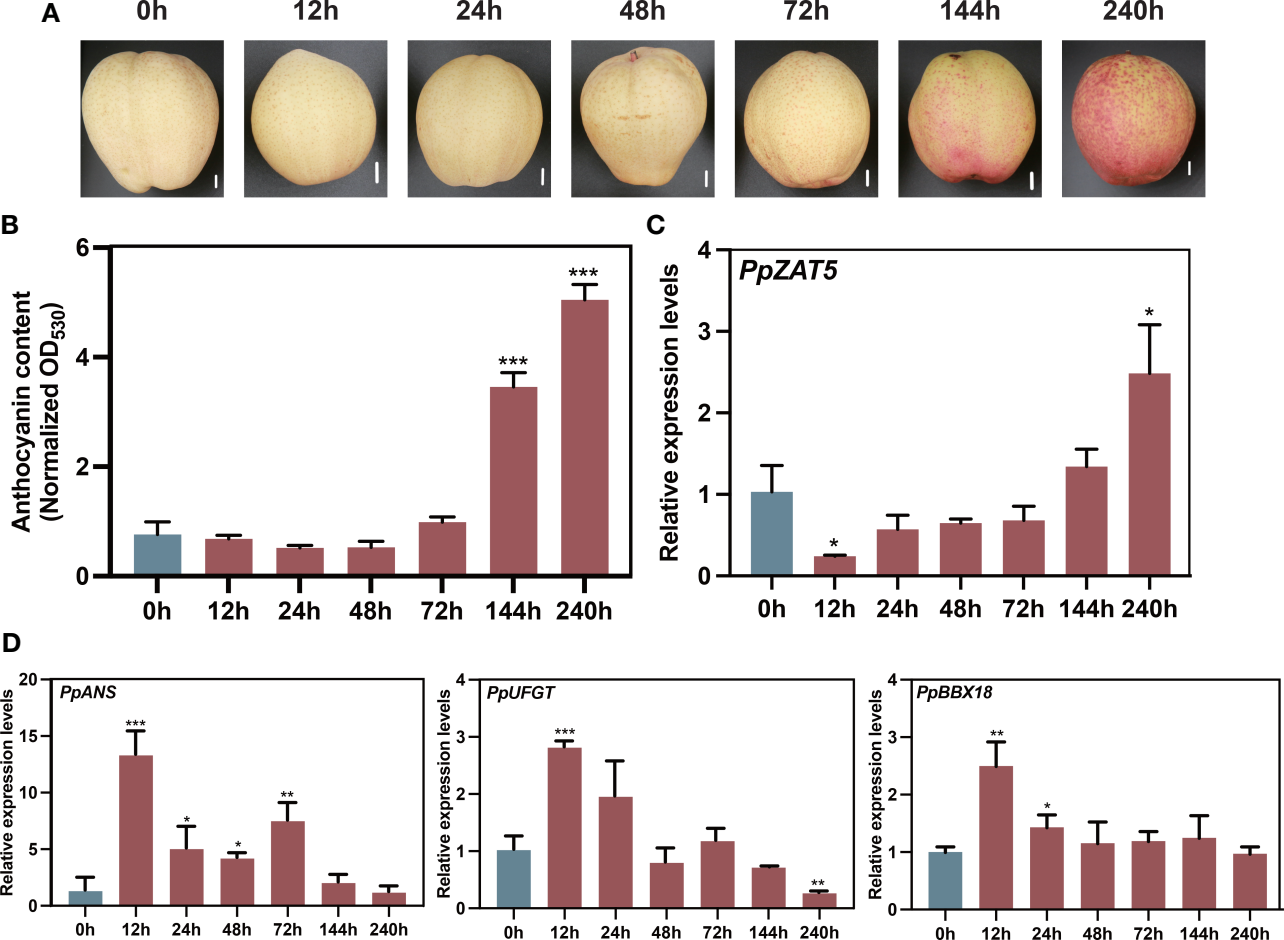
Light-responsive *PpZAT5* expression. **(A)** Anthocyanin accumulation in the pear, ‘Hongzaosu’ fruit peel after the light treatment. Scale bars = 1 cm. **(B)** Changes in the fruit peel anthocyanin contents during the light treatment. **(C)** *PpZAT5* expression pattern during light treatment. **(D)** *PpANS*, *PpUFGT* expression patterns during light treatment. Error bars for anthocyanin contents and expression levels represent the standard deviation of three independent experiments. Asterisks indicate significant differences (two-tailed Student’s *t*-test, *P < 0.05, **P < 0.01, ***P < 0.001).

Our previous transcriptome data of wild-type pear calli treated under continuously light conditions (Premathilake et al., 2020) revealed the low expressions of *PpZAT5* after 12 and 48 h in contrast to the increased expressions of anthocyanin-related structural genes after 48 h (**Supplementary Figure 1**). To further explore the expression pattern of PpZAT5 under light conditions, the *PpZAT5* expression was assessed in *‘*Hongzaosu’ pear fruit under continuous light conditions for 240 h. The anthocyanin accumulation started after 72 h and was significantly enhanced until the end of the continuous light treatment (**Figures 2A, B**). Meanwhile, the expression levels of anthocyanin biosynthesis-related genes *PpUFGT*, *PpANS*, and *PpBBX18* peaked at 12 h after starting the light exposure and then down-regulated soon after 24 h (**Figure 2D**), which was opposite to the expression of *PpZAT5*. *PpZAT5* expression was down-regulated after 12 h from starting to expose continuous light and upregulated gradually after 24 h (**Figure 2C**). These results indicate that *PpZAT5* responses to the light and might be related to light-induced anthocyanin accumulation.

### PpZAT5 directly bound to the promoter of *PpBBX18* and repressed the expression

**Figure 3:**
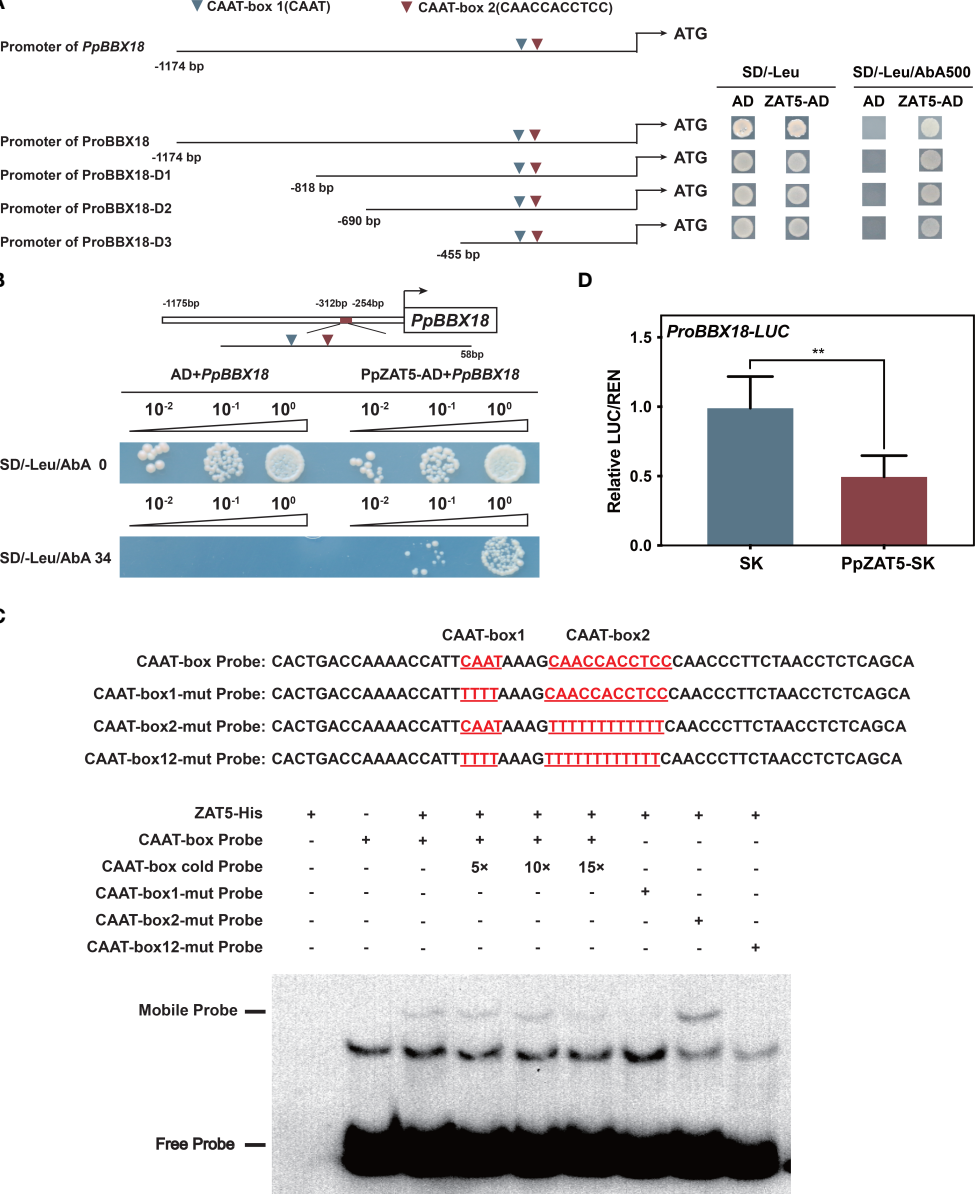
PpZAT5 was directly bound to the promoter of *PpBBX18* and repressed its expression. **(A)** Yeast one-hybrid assays of PpZAT5 on a series of fragments of the *PpBBX18* promoter. Red triangles indicate CAAT-box 1 (CAAT); Green triangles indicate CAAT-box 2 (CAACCACCTCC) **(B)** Yeast one-hybrid assays of PpZAT5 on a 58bp fragment of the *PpBBX18* promoter, which contains two CAAT-box. **(C)** Direct binding of PpZAT5 to CAAT-box1 of the *PpBBX18* promoter *in vitro*. **(D)** A dual-luciferase assay verified that PpZAT5 reduce *PpBBX18* promoter activity. Error bars for dual-luciferase assays represent the standard deviation of three independent experiments each with six technical replicates. Asterisks indicate significant differences (two-tailed Student’s *t*-test, **P < 0.01).

Given that PpZAT5 was discovered by yeast one-hybrid library screening assay with *PpBBX18* promoter as bait, we speculated that it might bind to the promoter of *PpBBX18*. To prove this speculation, four promoter fragments of *PpBBX18* (ranging from 1174 bp to 455 bp) were constructed for activity analysis. The results showed that PpZAT5 could bind to promoter fragments of *PpBBX18* from - 455 bp to 0 bp (**Figure 3A**). Then, we divided the 455 bp fragment into four parts, and the yeast one-hybrid assay result revealed that PpZAT5 could bind to the 58 bp DNA fragment from -312 bp to -254 bp (**Figure 3B**). Based on the 58 bp promoter fragment analysis, we found two CAAT-box motifs.

We further verified the binding activity in electrophoretic mobility shift assay (EMSA) with the 58 bp fragment as a probe. We found that the combination band of PpZAT5 protein and the biotin-labeled probe is getting fainter with adding the cold probes, which confirmed that PpZAT5 could bind directly to the *PpBBX18* promoter (**Figure 3C**). In addition, the PpZAT5 protein failed to bind to the *PpBBX18* promoter with a mutation to CAAT-box 1 or both CAAT-box 1 and CAAT-box 2, but mutation to CAAT-box 2 could not influence the binding between them (**Figure 3C**), which verified that PpZAT5 protein could bind to CAAT-box 1 of *PpBBX18* promoter. Furthermore, dual-luciferase assays showed that PpZAT5 significantly diminished transcriptional activation activity of PpBBX18 in contrast to the control, which demonstrated that PpZAT5 could repress *PpBBX18* expression (**Figure 3D**). To sum up, PpZAT5 could bind to CAAT-box 1 of the *PpBBX18* promoter directly and then inhibit the transcriptional activation activity of *PpBBX18*.

### Involvement of PpZAT5 in the regulation of anthocyanin biosynthesis

**Figure 4:**
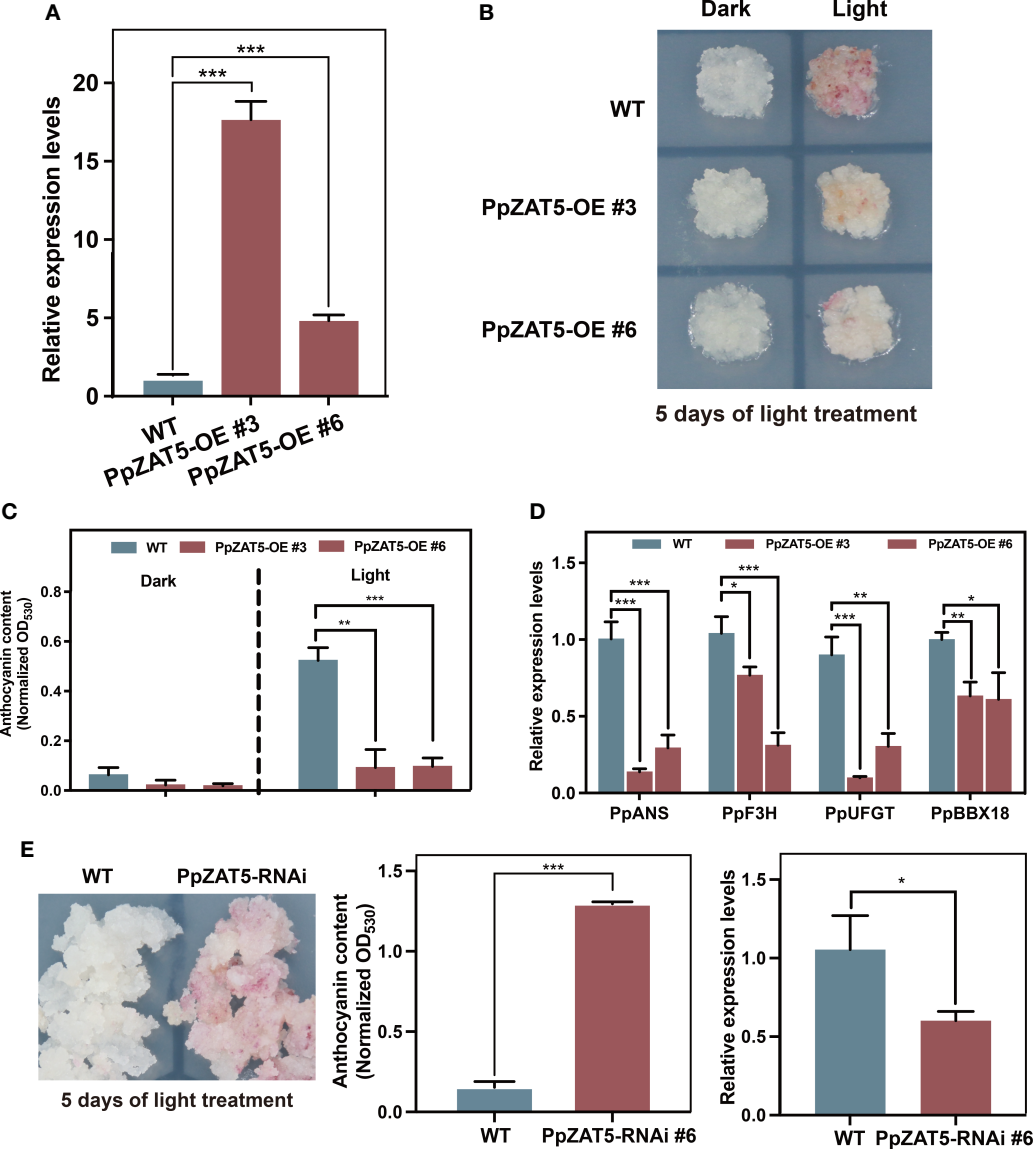
Overexpression and RNA interference (RNAi) of *PpZAT5* in pear calli. **(A)** The expression level of *PpZAT5* in overexpression pear calli. **(B)** Less anthocyanin accumulate in *PpZAT5-OE* pear calli. The pear calli were incubated under strong light at 17°C for 5 days. **(C)** Anthocyanin contents in *PpZAT5-OE* pear calli under dark and light treatment. **(D)** Expression levels of anthocyanin-related genes in pear calli under light treatment. **(E)** RNA interference (RNAi) of *PpZAT5* promotes anthocyanin accumulation in pear calli. Error bars for anthocyanin contents and expression levels represent the standard deviation of three independent experiments. Asterisks indicate significant differences (two-tailed Student’s *t*-test, *P < 0.05, **P < 0.01, ***P < 0.001).

To explore the function of PpZAT5 in anthocyanin biosynthesis, we generated a construct harboring the full-length coding sequence (CDS) of PpZAT5 under the control of the cauliflower mosaic virus 35S promoter (35S: PpZAT5). The construct was transformed into pear calli, while the wild-type pear calli were used as a control. Quantitative RT-PCR analysis showed that the expression levels of *PpZAT5* were significantly high in transgenic calli lines, PpZAT5-OE #3, #6 (**Figure 4A**). Both fresh wild-type pear calli and *PpZAT5*-expressing transgenic calli lines were treated in continuous light conditions until they turned red after 5 days. We found that lower-level of anthocyanin accumulated in *PpZAT5*-expressing transgenic calli lines compared to wild-type pear calli after 5 days of light treatment and under dark, no anthocyanin was accumulated in both wild-type and overexpressed lines (**Figures 4B, C**). Meanwhile, we found that compared to wild-type pear calli, the expressions of anthocyanin biosynthesis structural genes *PpF3H*, *PpANS*, and *PpUFGT* and light-responsive *PpBBX18* were endogenously down-regulated in PpZAT5-OE #3, #6 calli lines in contrast to wild-type calli (**Figure 4D**).

To validate, we also generated *PpZAT5-*RNA interference (RNAi) pear calli. In response to 5 days of continuous light treatment, more anthocyanin accumulated in the *PpZAT5-*RNAi than in the wild-type pear calli (**Figure 4E**). Moreover, to further prove the function of *PpZAT5* in anthocyanin biosynthesis, we generated *PpZAT5-*overexpressing pear calli with the different expression vectors. The results showed that *PpZAT5-*overexpressing pear calli with expression vector pCAMBIA1301or pCAMBIA1300 both accumulated less anthocyanin than wild-type after 5 days of continuous light treatment (**Supplementary Figure 2**). Taken together, the results indicate that *PpZAT5* regulates anthocyanin biosynthesis by inhibiting *PpBBX18* transcription.

### Transcriptome analysis of *PpZAT5* overexpression pear calli

**Figure 5:**
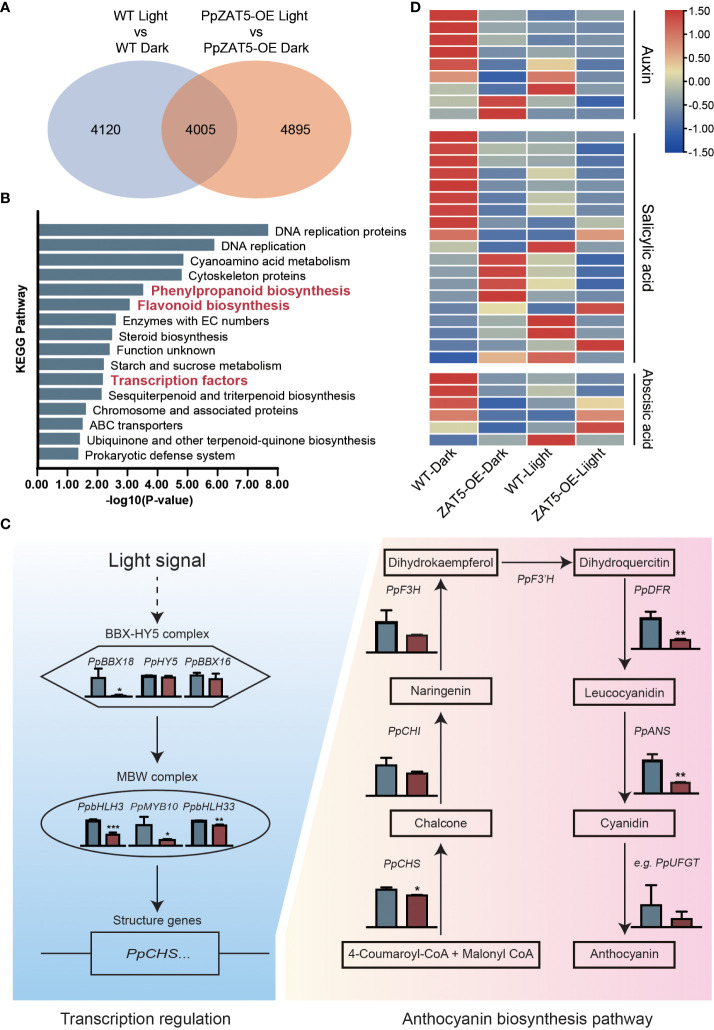
RNA-Seq analysis of *PpZAT5* overexpression calli under light conditions. **(A)** The Venn graph of the numbers of differential expression genes between wild-type and *PpZAT5* overexpression calli under dark/light conditions. **(B)** The enrichment analysis of KEGG pathways in the differential expression genes between wild-type and *PpZAT5* overexpression calli under light condition. **(C)** FPKM value of genes involved in light-induced anthocyanin biosynthesis in wild-type and *PpZAT5*-OE pear calli under light treatment. Blue color represents wild-type pear calli, red color represents *PpZAT5* overexpression pear calli. **(D)** The heatmap of differential expression genes involved in the phytohormone pathway between wild-type and *PpZAT5* overexpression calli under dark and light conditions. Error bars for the FPKM value represent the standard deviation of three independent experiments. Asterisks indicate significant differences (two-tailed Student’s *t*-test, *P < 0.05, **P < 0.01, ***P < 0.001).

To further analyze the function of *PpZAT5*, transgenic calli (35S: PpZAT5) and wild-type calli were treated under dark and continuous light conditions for 5 days. Then the wild-type and *PpZAT5*-overexpression calli were subjected to RNA-Seq analysis. The results showed that light treatment induced the transcription changes of more than 8000 genes, among which 4005 genes overlapped with *PpZAT5*-overexpression calli samples. On the other hand, 4895 genes were identified as light-induced differentially expressed genes in *PpZAT5*-overexpression calli specifically, which might be induced by light depending on *PpZAT5* (**Figure 5A**). The KEGG enrichment analysis was carried out in the differential expression genes of wild-type and *PpZAT5*-overexpression calli under light treatment. The results showed that related biological-related pathways of anthocyanin biosynthesis had relatively high P-values, such as flavonoid biosynthesis pathway, phenylpropanoid biosynthesis pathway, transcription factors, and others (**Figure 5B**). In addition, the crucial genes involved in light-induced anthocyanin biosynthesis, including transcription factors such as *PpBBX18, PpBBX16, PpbHLH3, PpbHLH33, PpMYB10*, and structure genes such as *PpCHS, PpCHI, PpF3H, PpDFR, PpANS* were markedly down-regulated in the transcriptome of *PpZAT5* overexpression pear calli comparing to wild-type (**Figure 5C**; **Supplementary Table 1**). In addition, transcriptome analysis showed differentially-expressed genes (DEGs) involved in the phytohormone pathway, including auxin, salicylic acid, and abscisic acid. Most of the DEGs were upregulated in *PpZAT5* overexpressed pear calli under dark conditions, but no differences were found between wild-type and *PpZAT5* overexpressed pear calli under light conditions (**Figure 5D**). These results further confirmed that *PpZAT5* was involved in the anthocyanin biosynthesis pathways and might also regulate the phytohormone pathway/metabolomics.

## Discussion

### PpZAT5 suppresses anthocyanin biosynthesis

**Figure 6:**
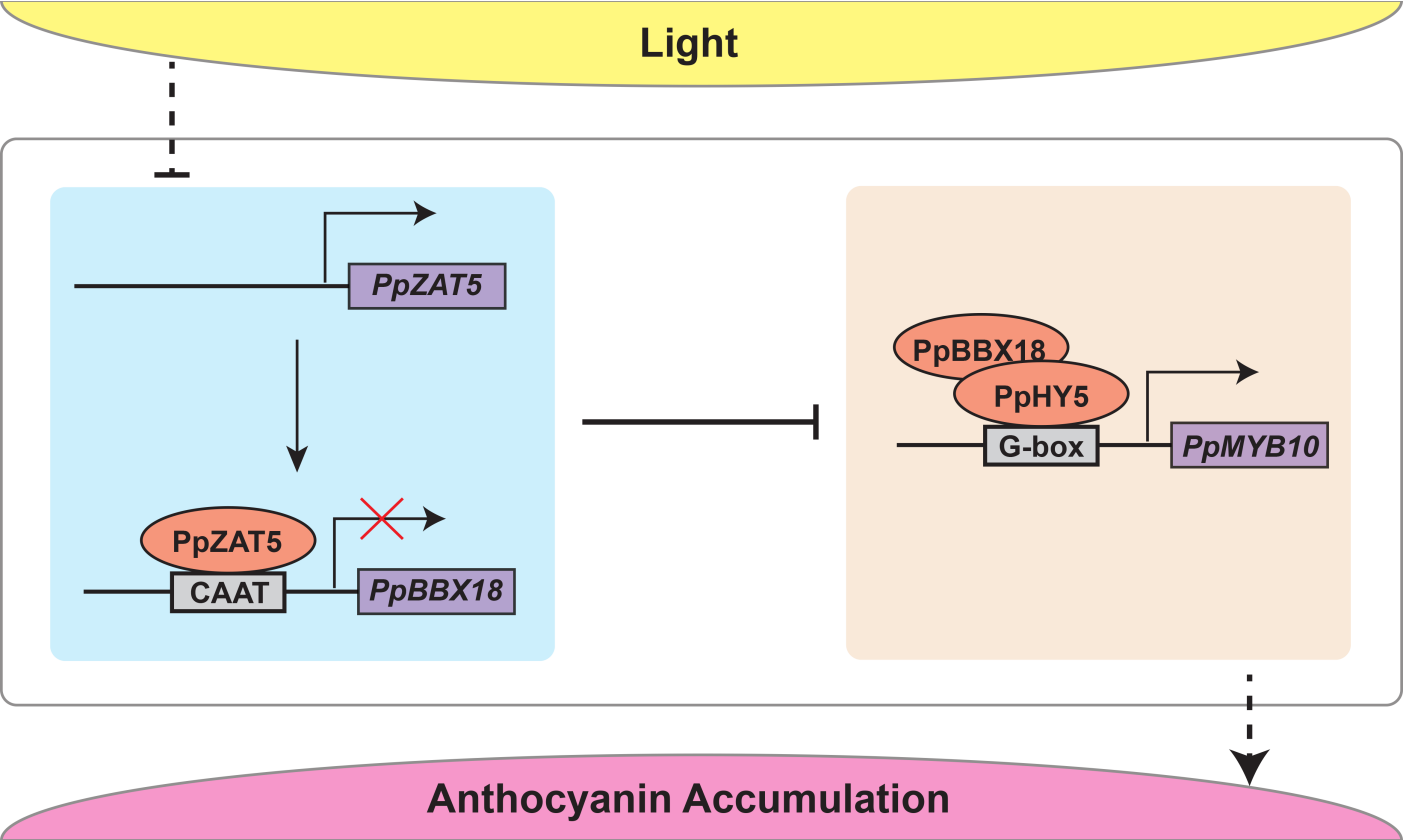
The proposed model indicates that under continuous light treatment, PpZAT5 downregulates the expression of *PpBBX18* by directly bound to its promoter and ultimately represses the signaling of light-induced anthocyanin accumulation in pear.

Generally, C2H2 zinc finger proteins act as positive regulators in anthocyanin biosynthesis and respond to abiotic stress (Englbrecht et al., 2004; Ciftci-Yilmaz and Mittler, 2008). In Arabidopsis, *SlZF2* (the ortholog of Arabidopsis ZAT10) was rapidly induced by abiotic stresses and overexpression of SlZF2 promoted anthocyanin accumulation (Hichri et al., 2014). *AtZAT6* responding to hydrogen peroxide (H_2_O_2_), positively regulated oxidative stress-induced anthocyanin and total flavonoid biosynthesis (Shi et al., 2018). A recent study showed that MdZAT5 responded to various abiotic stresses and promoted anthocyanin biosynthesis by increasing the expressions of anthocyanin-related genes (Wang et al., 2022). Like AtZAT5, the genes mentioned above and *PpZAT5* all belong to subgroup Cl-2i of C2H2 zinc finger proteins. However, our study showed that PpZAT5 functions as a transcriptional repressor to inhibit anthocyanin accumulation in pear (**Figure 6**). The phylogenetic analysis and multiple sequence alignment showed that *MdZAT5* (MD03G1128800) is the homolog of another pear gene (EVM0039484.1), not *PpZAT5* (EVM0021417.1) (**Figures 1A, B**). This gene is located on chromosome 3, while *PpZAT5* is on chromosome 11 of pear (*Pyrus pyrifolia*). Functional verification tests revealed the inhibitory effects of PpZAT5 on anthocyanin biosynthesis in PpZAT5-overexpression and RNAi pear calli (**Figure 4**). Transcriptome analysis of the wild-type and *PpZAT5*-OE pear calli indicated that PpZAT5 modulates the expression of TF genes related to light-induced anthocyanin biosynthesis and structural genes of anthocyanin biosynthesis passway (**Figure 5C**). The inhibitory effect of PpZAT5 on anthocyanin accumulation might contribute to the counter-balancing mechanism of fine-tuning anthocyanin biosynthesis by cooperating with other positive regulators, which enriches the network of light-regulated anthocyanin biosynthesis.

### Light promotes anthocyanin biosynthesis by inhibiting the expression of PpZAT5, a transcription repressor

The process of anthocyanin biosynthesis is fine-tuned. At present, the repressor MYB proteins and some BBX proteins have been studied a lot, they antagonize with positive regulators and fine-tune anthocyanin accumulation. In our study, the *PpZAT5* expression pattern is converse to anthocyanin-related genes. When transferred to light conditions from dark, *PpZAT5* expression was repressed at first and recovered gradually (**Figure 2C**), while anthocyanin-related genes were strongly activated early and gradually decreased (**Figure 2D**). Taking account of the interaction between PpZAT5 and *PpBBX18* as well as their function, we speculated that the inhibitory effect of PpZAT5 on anthocyanin biosynthesis might be in the late period of light treatment and PpZAT5 is a braking gene in the process of anthocyanin biosynthesis.

*PpZAT5* expression was repressed by light (**Figure 2C**), which disinhibited the effect on *PpBBX18* and promoted anthocyanin biosynthesis. Interestingly, light inhibition on transcription repressors has not been reported, but inhibition of light on transcription activators is universal. In Arabidopsis, HDA19 and SNLs are the crucial negative regulators of the light signaling pathway, and their expressions are repressed by light (Jing et al., 2021). BR is one of the important inhibitory factors of photomorphogenesis, and light suppresses the expression of key BR biosynthesis genes. Their expressions were significantly lower in light-grown seedlings than in seedlings grown in the dark (Zhang et al., 2021). Light signal inhibits the DNA binding ability of ARF6 and ARF8 by regulating direct interaction between photoreceptor CRY1and phyB with ARF6 and ARF8. In addition, light signal inhibits the transcriptional activation of ARF by promoting the accumulation of Aux/IAA protein and the interaction between Aux/IAA and ARF to inhibit auxin signal, thus promoting photomorphogenesis (Mao et al., 2020). In our study, light repressed the expression of *PpZAT5*, and transcriptome analysis of *PpZAT5* overexpression pear calli showed that it might participate in the phytohormone pathway, including auxin, salicylic acid, and abscisic acid, speculated that *PpZAT5* might be repressed by light similarly.

### PpZAT5 with EAR motif may act as a transcription repressor

Phylogenetic analysis showed that PpZAT5 is the homolog of Arabidopsis AtZAT5; meanwhile, multiple sequence alignment showed that PpZAT5 possesses two dispersed C2H2-type zinc fingers and an EAR motif related to repression activity (**Figures 1A, B**). EAR (ERF-associated amphiphilic repression) motif is the first active repressing motif found in plants and is in the C-terminal region of genes. It is the most representative transcriptional repression motif in plants (Ohta et al., 2001; Kagale et al., 2010). The repression capacity of NtERF3, an ERF protein from tobacco, is completely abolished when deleted C-terminal EAR motif region. Likewise, the deletion of C-terminal EAR motif regions eliminates the inhibitory effect of ZAT10 and ZAT11 on downstream genes in Arabidopsis (Ohta et al., 2001). A subset of zinc finger proteins (ZFPs) with EAR motifs have been identified as active repressors in various plant species (Kagale et al., 2010). Transcription repressors of C2H2-type zinc finger proteins containing EAR-motif at the C terminus act as a crucial negative regulator in the growth and development of plants (Singh et al., 2019). In Arabidopsis, *AtZAT10* is induced rapidly and transiently by cold and then represses a classical stress-response gene *RD29A* transcription by binding to its promoter (Lee et al., 2002). *AtZAT12* downregulated the expression of the CBF genes that play significant roles in the cold response pathway, indicating it is involved in a negative regulatory circuit to regulate cold tolerance of Arabidopsis (Vogel et al., 2004). Moreover, *AtZAT12* loss-of-function lines are more resistant to heat stress, demonstrating that AtZAT12 functions as a repressor to influence the heat resistance of Arabidopsis (Davletova et al., 2005). In our study, PpZAT5 was localized at nuclear and showed strong trans-repression activity in yeast compared to the positive control VP16 (**Figures 1C, D**), which confirmed it functions as a transcription repressor in plant physiological metabolism. The transcriptional repressing activity of PpZAT5 might be due to the EAR motif at the C-terminal region.

### PpZAT5 represses the expression of *PpBBX18* by binding to the CAAT motif

Both internal and external factors influence anthocyanin biosynthesis in plants, and light was thought to be a vital external factor for anthocyanin biosynthesis (Sun et al., 2014; Bulgakov et al., 2017). A series of BBX proteins have been confirmed to regulate photomorphogenesis and light-induced anthocyanin biosynthesis pathways (Holtan et al., 2011; Xu et al., 2016; Zhang et al., 2017; Job et al., 2018). According to the number of B-box and CCT domains, BBX proteins can be divided into five types, from I to V in Arabidopsis (Khanna et al., 2009). Most BBX proteins of type IV are involved in HY5-dependent photomorphogenesis. HY5 has been shown to functionally interact with B-box-containing proteins such as BBX21-BBX22 and BBX24-BBX25 in Arabidopsis. BBX21 and BBX22 promote HY5 function in photomorphogenesis by binding to its promoter directly, while BBX24 and BBX25 suppress photomorphogenesis through direct physical interaction (Gangappa and Botto, 2016). Our previous study has proved that PpBBX18 could provide the *trans-acting* activity for PpHY5 as a result, positively regulated anthocyanin biosynthesis in pear (Bai et al., 2019b). In our study, dual-luciferase assays and yeast one-hybrid deletion assay showed that PpZAT5 repressed the expression of *PpBBX18* by binding to a 58bp DNA fragment of *PpBBX18* promoter (**Figures 3A, B, D**). Therefore, overexpression of PpZAT5 in pear calli inhibited anthocyanin accumulation while PpZAT5-RNAi pear calli accumulated more anthocyanin (**Figure 4**). These results indicate that PpZAT5 with the EAR motif suppresses anthocyanin biosynthesis by repressing the expression of *PpBBX18*.

The recognition mechanism of C2H2 zinc finger proteins to target DNA is not thoroughly studied. The function of C2H2 zinc finger proteins in regulating downstream genes expression began with the ZPT2-2 identified in petunia, and ZPT2-type proteins recognized two tandemly repeated AGT sequences separated by about 10 bp (Takatsuji et al., 1992; Takatsuji, 1998). AtZAT10 recognizes AG(C)T sequences separated by 3 bp or 4 bp (Sakamoto et al., 2004). In Arabidopsis, some C2H2-type zinc finger proteins bind to TACA(A/G)T element that is different from ZPT2-type proteins. Di19 (one C2H2 zinc-finger protein) could promote the expression of *PR1, PR2*, and *PR5* by binding to the TACA(A/G)T element of their promoters, resulting in increased drought tolerance of Arabidopsis (Liu et al., 2013). AtZAT6 also binds to the TACAAT element of downstream gene promoters such as *EDS1, PAD4, PR1, PR2*, and *PR5* and then increases their expression to modulate the biotic and abiotic stress responses (Shi et al., 2014). In our study, the electrophoretic mobility shift assay (EMSA) verified PpZAT5 binding to the CAAT-box 1 (CAAT) motif of the *PpBBX18* promoter (**Figure 3C**). Compared to TACA(A/G)T, CAAT-box 1 (CAAT) has two fewer bases, which verified that the CAAT motif might be the core site of PpZAT5 binding to the *PpBBX18* promoter. Our study gives possibilities for C2H2-type zinc finger proteins to regulate more potential downstream genes.

In a word, our previous studies have shown that PpBBX18 interacts with PpHY5 *via* two B-box domains and then forms a heterodimer, in which PpHY5 binds to the G-box motif of *PpMYB10* and PpBBX18 provides the *trans*-acting activity, thus inducing transcription of *PpMYB10* as well as anthocyanin biosynthesis (Bai et al., 2019b). In this study, we found that PpZAT5 inhibits anthocyanin biosynthesis by binding to CAAT motif of *PpBBX18* promotor and then represses its expression (**Figure 6**).

## Data availability statement

The datasets presented in this study are publicly available. This data can be found with the accession No. PRJNA871954 in SRA database of NCBI.

## Author contributions

SB and YT conceived and supervised the research work. LZ performed most experiments and wrote the manuscript. RT started the experiments. SW helped to revise the manuscript. YG helped to analyze the RNA-Seq data. LW, SY, XZ, WY, XW, KL provided technical assistance. JN helped to write the manuscript. All authors contributed to the article and approved the submitted version.

## Funding

This research was supported by Zhejiang Provincial Natural Science Foundation of China under Grant No. LR22C150001, the National Natural Science Foundation of China (32072545 to YT), and the China Agriculture Research System of MOF and MARA.

## Conflict of interest

The authors declare that the research was conducted in the absence of any commercial or financial relationships that could be construed as a potential conflict of interest.

## Publisher’s note

All claims expressed in this article are solely those of the authors and do not necessarily represent those of their affiliated organizations, or those of the publisher, the editors and the reviewers. Any product that may be evaluated in this article, or claim that may be made by its manufacturer, is not guaranteed or endorsed by the publisher.
