# Supplementary material for: PpZAT5 suppresses the expression of a B-box gene PpBBX18 to inhibit anthocyanin biosynthesis in the fruit peel of red pear
Source: Front Plant Sci. 2022 Oct 11;13:1022034. doi: 10.3389/fpls.2022.1022034 (PMC9592862; doi:10.3389/fpls.2022.1022034)
Supplement: Supplementary file 1 [file DataSheet_1.pdf]

Supplementary Material

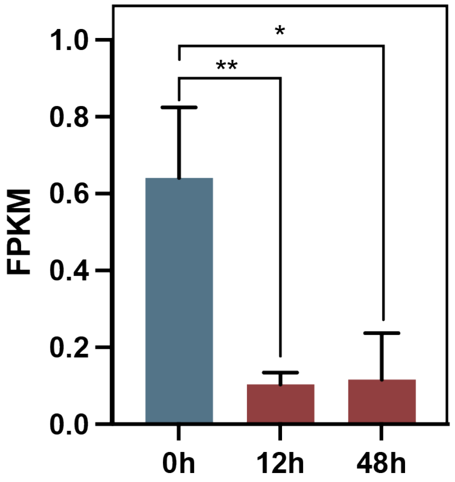

Supplementary Figure 1. FPKM value of PpZAT5 in the pear calli under light treatment. The transcriptome data were reported previously (Premathilake et al., 2020). Error bars for the FPKM value represent the standard deviation of three independent experiments. Asterisks indicate significant differences (two-tailed Student's *t*-test, * $P < 0.05$, ** $P < 0.01$, *** $P < 0.001$).

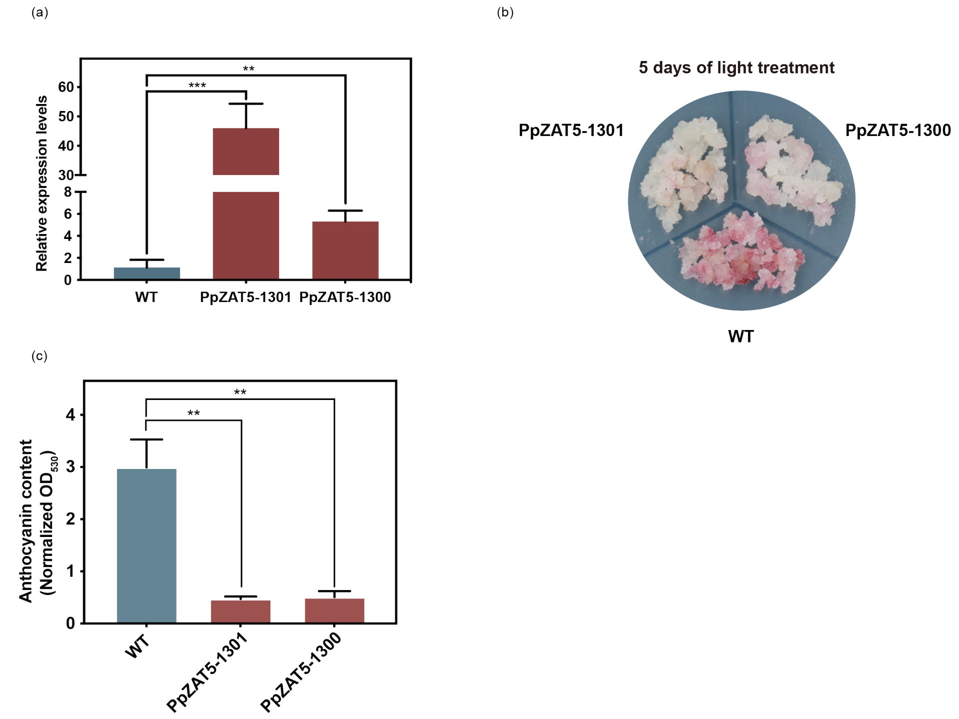

Supplementary Figure 2. Overexpression of *PpZAT5* with different vectors in pear calli. (a) Expression levels of *PpZAT5* in pear calli under light treatment. (b) Overexpression of *PpZAT5* inhibited anthocyanin accumulation in pear calli. The pear calli were incubated under strong light at 17°C for 4 days. (c) Anthocyanin contents in *PpZAT5-OE* pear calli under light treatment. Error bars for anthocyanin contents and expression levels represent the standard deviation of three independent experiments. Asterisks indicate significant differences (two-tailed Student's *t*-test, * $P < 0.05$, ** $P < 0.01$, *** $P < 0.001$)

Supplementary Table 1

FPKM average value in wild type and PpZAT5-OE pear calli transcriptome

	wild type	PpZAT5-OE
PpHY5	27.30	25.67
PpBBX18	0.88	0.05
PpBBX16	2.76	2.28
PpbHLH3	7.46	3.34
PpbHLH33	24.57	20.19
PpMYB10	2.34	0.65
PpCHS	202.17	134.16
PpCHI	17.41	12.79
PpF3H	173.20	98.33
PpDFR	99.25	29.10
PpANS	376.42	123.23
PpUFGT	0.27	0.10

Supplementary Table 2

The primers used in the present work

Primer Names	Forward(5'-3')	Reverse(5'-3')	Usage
PpZAT5	ATGGAGGGCCAAGAAGAATT	TTAATACTGACAATCCACCAA	Vector construction
ProPpBBX18	TAGTGTGTGGAGTAGCAGGG	CCCTTAGATTTAGCTTCTCT	Vector construction
Q-PpZAT5	TGCTACACATGTGGCTGTGA	GGGTAGTTTGTTGGGTAGGCA	qRT-PCR
Q-PpBBX18	AATGCCCCGTTTGGTTTCTC	CCACATTCTGTTGCTGGCTT	qRT-PCR
Q-PpF3H	GGAGAAAGACAAAGTGGAGATAAAGC	ACAAGAAGTGGAAAGGCAAAGTTAC	qRT-PCR
Q-PpANS	AGTTGTTCAGGAAAAGCCAAGAGG	ACAAAGCAGGCAGATAGGAGTAGC	qRT-PCR
Q-PpUFGT	CTGGAACCTGAAGTTGTGAATCTG	AGCCACTCTAAGCAACCACTATC	qRT-PCR
